# Inhalation exposure to dihydroxyacetone promotes lung injury and pulmonary fibrosis in A/J mice

**DOI:** 10.1016/j.toxrep.2024.101878

**Published:** 2024-12-18

**Authors:** Arlet Hernandez, Hailey Levi, Juan Xavier Masjoan Juncos, Lilly Underwood, Jenna Hedlich-Dwyer, Saurabh Aggarwal, Natalie R. Gassman

**Affiliations:** a Department of Pharmacology and Toxicology, The University of Alabama at Birmingham, 1720 2nd Ave S, Birmingham, AL 35294, USA; b Department of Anesthesiology and Perioperative Medicine, The University of Alabama at Birmingham, 901 19th ST S, Birmingham, AL 35294, USA; c Department of Cellular and Molecular Medicine, Florida International University, AHC-1 418C, 11200 SW 8th street, Miami, FL 33199, USA

**Keywords:** E-cigarette, Lung, Inhalation, DHA, Pulmonary

## Abstract

Acute and sub-acute exposure to dihydroxyacetone (DHA), a compound found in e-cigarette aerosols and spray tanning products, was assessed for its impact on lung injury in A/J mice. Mice were exposed to inhaled DHA doses of 5, 130, and 600 µg and evaluated at 1 and 24 h post-exposure. Acute exposure to DHA led to significant inflammatory responses, indicated by increased bronchoalveolar lavage fluid (BALF) protein levels at 5, 130, and 600 µg and notably elevated inflammatory cytokines IL-6 and TNF-α 1 h post-exposure. 24 h post-exposure, the 130 and 600 µg doses showed elevated BALF cell counts. Histological analysis revealed significant alveolar damage and increased lung injury scores for the 130 and 600 µg doses. For sub-acute exposure, male and female mice were exposed to 5 µg DHA for 14 days. Increased BALF cell counts and protein levels were observed, with sex-specific differences in cytokine responses. Male mice exhibited reduced levels of IFN-γ and TNF-α, while female mice showed significant lung damage characterized by decreased alveolar density and increased collagen deposition indicative of fibrosis. Functional assessments showed mixed obstructive and restrictive lung changes. This study highlights DHA’s potential to induce acute inflammatory responses and chronic lung damage, including both emphysematous and fibrotic changes. These findings suggest that DHA exposure, particularly from aerosolized e-liquids, could contribute to respiratory complications, underscoring the need for further research on long-term exposure effects.

## Highlights

• Dihydroxyacetone (DHA) inhalation produces acute and sub-chronic lung injury.
• Acute and sub-chronic DHA exposure induce inflammatory cytokines.
• E-cigarette relevant quantities of DHA induce obstructive and restrictive phenotypes and reduced lung function.

## Graphical Abstract

**Figure:**
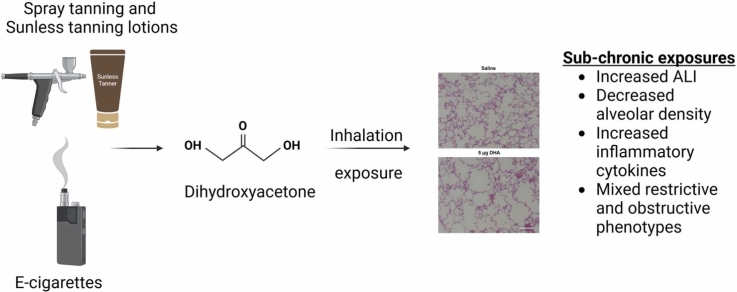

## 1 Introduction

Electronic cigarettes (e-cigarettes), introduced in 2007, have become increasingly popular over the past decade, particularly among young adults. Despite various studies demonstrating adverse effects after vaping, over 2 million teens and 8.1 million adults are current e-cigarette users [1], [2], [3]. E-cigarette devices come in a variety of sizes, may be battery-operated, and are offered with various e-liquids containing flavorings and/or nicotine. E-liquids are composed of two main ingredients, propylene glycol and vegetable glycerol, that are heated to create the aerosol the user inhales [4], [5]. While significant emphasis has been placed on the dangers of nicotine and flavorings in e-cigarettes, few studies have examined the effects of specific combustion products generated by e-liquids. E-liquids can generate unexpected chemicals, many of which are toxic [6], [7], [8]. Propylene glycol and vegetable glycerol (PG: VG) inhalation studies have shown increased inflammation, oxidative stress, and altered lung mechanics in the absence of additional ingredients [6], [7], [9], [10], [11], [12], [13], [14]. Increases in bronchial alveolar lavage fluid (BALF) cell count were observed after 3 days and 4 weeks of PG:VG 1:1 exposure in male C57BL/6 mice. Resistance and elastance changes were also observed after 3 days of exposure, altering lung mechanics and airway responsiveness [15]. Despite similarities in these findings with various types of e-cigarette devices and PG:VG ratios, there are inconsistencies in the culprit attributed to the effects [13], [16], [17].

One overlooked component of e-cigarette aerosol is dihydroxyacetone (DHA), a triose sugar produced by glycerol oxidation [8]. DHA is produced at ∼0.16–4.16 µg per puff in a 100 ml puff volume, dependent on the device’s wattage, making it a high-concentration component of e-cigarette aerosol [8]. Given that DHA exposure amounts have not been quantified, we can estimate exposure based on e-cigarette user studies [8], [18]. Puff volumes can vary from 96.81 to 133.92 ml per puff for cigarette-like devices to 331.2–519.6 ml for tank devices [18], [19]. Most studies suggest a 10-puff per session duration with up to 24 smoking sessions per day for vapers. Therefore, DHA exposures range from 38.4 µg/day to ∼ 1 mg/day, allowing a user to potentially inhale high micromolar to low millimolar doses daily [8], [18], [19].

In addition to inhalation exposure from e-cigarettes, DHA is also the active ingredient in sunless tanning products (STPs), including spray tans [4], [20]. The European Union Regulatory Commission calculated inhalation exposures to DHA in spray tanning booths from 0.21 to 0.6 mg per session [21]. Although DHA has been approved for topical use in STPs by the FDA at concentrations up to 20 %, inhalation of DHA has been warned against, and inhalation exposure effects are currently unknown [22], [23].

*In vitro* studies across various cell models demonstrated DHA induces cytotoxicity, genotoxicity, metabolic imbalances, oxidative stress, and mitochondrial dysfunction [20], [24], [25], [26], [27], [28], [29], [30]. Some of the characterized DHA effects overlap with those found in PG:VG exposures, suggesting DHA may contribute to the adverse inhalation effects observed. No study has characterized the inhalation effects of DHA to understand its potential exposure effects. Therefore, we have assessed inhaled DHA exposure using A/J mice. This model is widely used for evaluating lung inflammation in toxicological studies of traditional cigarette smoke [31].

## 2 Materials and methods

### 2.1 *In vivo* exposure to DHA

All animal procedures were approved by the University of Alabama at Birmingham Institutional Animal Care and Use Committee (IACUC), protocol 22789. Un-anesthetized A/J mice were exposed to DHA (PHR 1430, Sigma Aldrich, St. Louis, MO, US) dissolved in saline or saline control using the Kent Scientific Aeroneb Lab Control Module with a nose-only animal holder (Kent Scientific, Torrington, CT, US). Male A/J mice were exposed to 5, 130, or 600 µg DHA for acute exposure of 5 min, and acute lung injury was measured 1 h or 1 day post-exposure. These doses were selected based on DHA production volumes (∼0.16–4.16 µg per puff in a 100 ml puff) and reported vaping behaviors to examine estimated low, moderate, and high doses [8], [18], [19]. To determine the role of sub-acute exposure, male and female A/J mice were exposed to 5 µg of DHA 6 days on and 1 day off for 2 weeks, and parameters of lung injury were measured. After exposures, mice were returned to room air and monitored continuously for signs of distress. Body weight and blood glucose were measured throughout the exposure period.

### 2.2 Bronchial alveolar lavage fluid (BALF) measurements

Male A/J mice were euthanized, and an incision was made at the neck to expose the trachea and insert an endotracheal cannula. The lungs were lavaged by pushing 1 ml of PBS through the cannula 2–3 times to collect the bronchial alveolar lavage fluid (BALF). The recovered fluid was placed on ice and centrifuged at 3000× g for 10 min to pellet the cells. Protein concentrations were measured using the supernatant and quantified with a BCA Protein Assay Kit. The cells were resuspended in 100 µL pf PBS and counted using a hemocytometer. Cell and protein count values were graphed using GraphPad Prism and displayed as mean ± SD. Significance for the acute exposures were calculated using a one-way analysis of variance with Dunnett’s post hoc test and a Welch’s *t*-test for the sub-acute exposures.

### 2.3 Cytokines analysis

V-PLEX Proinflammatory panel from Meso Scale Diagnostics (MSD, Rockville, MD, US) was used to perform cytokine analysis. Blood from A/J male mice exposed to DHA at acute doses 5, 130, and 600 µg for 5 min was collected 1 and 24 h post-exposure. In addition, blood was collected from A/J female and male mice sub-acutely exposed to DHA for 2 weeks using K_2_EDTA blood collection microtainer tubes (BD Microtainer Beckton Edison, Franklin Lakes, NJ, US). Blood was inverted gently at least 10 times, then centrifuged at 2000× g for 10 min, and plasma was collected. A cytokine panel including interferon-gamma (IFN-g), interleukin-1 beta (IL-1β), interleukin-2 (IL-2), interleukin-5 (IL-5), interleukin-6 (IL-6), interleukin-10 (IL-10), interleukin-12 (IL-12p70), keratinocyte chemoattractant/human growth regulated oncogene chemokine (KC/GRO) and tumor necrosis factor-alpha (TNF-α) was performed according to manufacturer’s protocol. The samples were diluted two-fold in diluent 41 provided by the kit. The samples, calibrators, and controls were added to the plate and incubated at room temperature (RT) for 2 h while shaking. Then, each well was washed three times with a wash buffer, and the antibody detection solution containing corresponding antibodies was added and incubated for another 2 h while shaking. After incubation, the plate was washed three times, and the read buffer was added to the wells. The plate was read using an MSD instrument, and the values were plotted using a heat map analysis or mean ± SD values in GraphPad Prism. The significance was calculated using a one-way analysis of variance with Dunnett’s post hoc test for acute exposures, a Welch’s *t*-test for the sub-acute exposures. Outliers were identified and removed using the ROUT method. We also analyzed the overall cytokine changes with a mixed-effects model. For acute exposure, the dose (p < 0.05) and dose x cytokines (p < 0.0001) were significant. For the sub-acute exposures, the males showed significant dose x cytokine effects (p < 0.01), and the females only showed no significant changes in dose or dose x cytokines.

### 2.4 Respiratory mechanics using FLEXIVENT

Male A/J mice from sub-acute exposures were placed on a mechanical ventilator and challenged with methacholine injections from 0 to 40 mg/ml to evaluate lung mechanics. First, they were anesthetized and paralyzed using pentobarbital, and a small incision in the trachea was performed to insert a cannula. The mice were then connected to an FX-1 module of the FLEXIVENT (SCIREQ, Paris, France). Increasing concentrations of methacholine chloride were administered via aerosolization, and airway responsiveness was recorded every 15 s for 3 min for each challenge. Several physiological parameters were measured and calculated for both control and DHA-treated mice. The pressure-volume (PV) curves show changes in obstructive and restrictive respiratory disease along with overall resistance of the respiratory system (R_RS_), overall compliance of the respiratory system (E_RS_), tissue damping (G), tissue elastance (H) and resistance (R_N_). The graph is displayed as mean ± SEM values for the variables calculated.

### 2.5 Immunohistochemistry

Acute lung injury and alveolar density were evaluated in lung sections in mice exposed to acute or sub-acute doses of DHA, as mentioned above. The tissues were fixed in Bouin’s solution, processed, embedded, and stained with hematoxylin and eosin (H&E) by UAB’s Comparative Pathology Lab. After staining, five different regions in the tissue slides were imaged using the all-in-one Keyence microscope with a 10X objective (NA 0.45). Lung sections were scored for each measurement. Acute lung injury was scored using five parameters: neutrophils in alveolar space, neutrophils in the interstitial space, development of hyaline membranes, debris in the airspace, and septal thickening, as outlined in Matute-Bello et al. [32]. A score for each image was obtained and averaged per mouse. Alveolar density was also evaluated in H&E-stained lung slides, and an alveolar space count was obtained per image. The values obtained were normalized to the image field size and averaged per mouse. Values are displayed as mean ± SD. Significance was calculated using a one-way analysis of variance with Dunnett’s post hoc test for the acute exposures and a Welch’s *t*-test for the sub-acute exposures.

Masson’s Tri-chrome stain was performed in the lungs to evaluate the density of collagen deposition. The tissues were fixed in Bouin’s solution and embedded into slides. Tissue sections were deparaffinized and treated with Bouin’s fluid at 56°C for 1 h, then stained with Weigert’s Iron Hematoxylin, Biebrich Scarlet-Acid Fuchsin solution, and Aniline Blue Stain Solution. 1 % Acetic Acid solution is then applied to remove any excess staining. After this process, collagen is stained blue, nuclei are stained black, and cytoplasm and muscle fibers are red. The whole lung tissue slides were imaged using an all-in-one Keyence microscope using the 10× objective. Collagen percent was quantified using a binary mask threshold of the whole lung image in ImageJ. The values obtained are displayed as mean ± SD. The significance was calculated using a Welch’s *t*-test.

### 2.6 Immunofluorescence

Immunofluorescence was performed on lungs from sub-acutely exposed A/J female mice to evaluate lung myeloperoxidase (MPO). Tissue sections were deparaffinized using three changes of xylene for 5 min each. Then, the tissues were re-hydrated in 100 %, 95 %, 70 %, and 50 % ethanol for 3 min each. Antigen retrieval was performed in antibody signal enhancer (ASE) buffer containing 10 mM glycine, 1 % Triton-X, and 0.05 % Tween-20 [33]. The slides were immersed in the ASE solution and heated three times with 18 s on and 5 min cooling off first, then 15 s on and 5 min cooling off twice. The slides were then cooled to RT, rinsed with Milli-Q H_2_O, and dried off. The slides were blocked with 5 % goat serum for 30 min, and the respective primary antibody was placed on slides: MPO (1:100 PA5–16672 Invitrogen) in 5 % goat serum overnight (ON). The next day, the slides were washed three times for 5 min each with 1X Tris-buffered (TBS) with 0.1 % Tween-20 (TBST). The slides were dried off and incubated for 1 h at RT with corresponding secondary antibodies AlexaFluor 546 (1:400 Thermo Fisher) diluted in 5 % goat serum. Nuclear staining was performed 15 min before incubation using 4’6-diamidino-2-phenylindole (DAPI) staining (1:400 Thermo Fisher). After incubation, the slides were washed three times with 1X TBST for 5 min. The slides were then mounted using Prolong Gold and allowed to dry ON. The whole lung slides were imaged using all-in-one Keyence using the 10× objective. The Nikon Elements software was used to perform the analysis. The fluorescent intensity was measured using a binary threshold over the whole tissue, and regions of interest (ROI) were selected throughout the image. The values obtained were graphed using GraphPad Prism and displayed as the mean ± SEM. Significance was calculated using a Welch’s *t*-test.

## 3 Results

### 3.1 Acute DHA exposure-induced lung injury

**Fig. 1:**
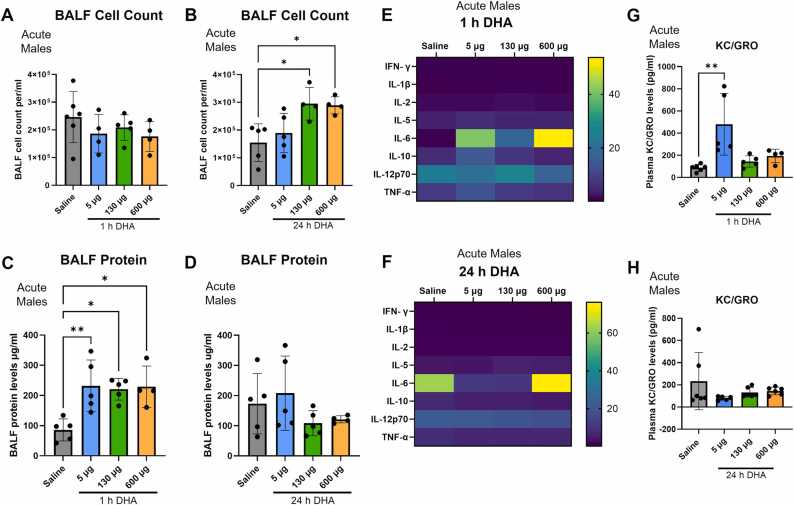
Acute exposure to DHA induced inflammation in male mice. Male A/J mice were exposed to 5, 130, and 600 µg of DHA and total cell count per ml in BALF was measured (A) 1 h or (B) 24 h after exposure. Protein concentrations were also measured in these mice at (C) 1 h and (D) 24 h after exposure. A V-PLEX Proinflammatory cytokine panel was performed in plasma after acute exposures. Cytokine levels after exposure to 5, 130, and 600 µg of DHA for 1 h (E) and 24 h DHA exposure (F) are displayed using a heat map. KC/GRO levels were measured at the 5, 130, and 600 µg doses for 1 h DHA (G) and 24 h DHA (H). Mean values are displayed as mean ± SD of values. Statistical significance was determined by ANOVA with significance is displayed as follows: *p < 0.05, **p < 0.01, ****p < 0.0001.

We first evaluated acute lung injury parameters 1 h or 1-day post-acute exposures to 5, 130, and 600 µg of DHA. Using the DHA aerosol measurements made by Vreeke et al., 0.16–4.16 µg per puff in a 100 ml puff consistent for a cigarette-like device, we estimate these users would inhale 1.6–41.6 µg of DHA in a single 10-puff vaping session with the possibility of multiple sessions per day [6], [8], [18], [19]. We estimated a 400 ml puff volume for the tank-like devices, resulting in 6.4–166.4 µg per 10-puff session with the possibility of multiple sessions per day. Therefore, 5 and 130 µg represent these ranges, though these doses are conservative in the number of sessions per day. The 600 µg dose was selected from the European Union estimates for spray tanning and would represent multiple daily vaping sessions [34]. Male A/J mice were acutely exposed to 5, 130, and 600 µg DHA for 5 min. BALF total inflammatory cell count and protein content were measured 1 and 24 h after exposure (Fig. 1). At 1 h post-DHA exposure, no change occurred in total cell count between dosing groups compared to control mice exposed to saline, while at 24 h, a significant increase was found post-exposure to 130 and 600 µg DHA (Fig. 1A and B). The analysis of BALF protein levels demonstrated a significant increase 1 h post-exposure in mice exposed to 5, 130, and 600 µg DHA (Fig. 1C). However, BALF protein levels at 24 h post-exposure returned to saline vehicle levels (Fig. 1D).

Plasma collected at 1 and 24 h post-exposure to 5, 130, and 600 µg of DHA was used to perform an inflammatory cytokine analysis (Fig. 1E-H). At 1 h post-exposure, no changes were measured in IFN-γ, IL-1β, IL-2, IL-5, and IL-12p70 levels at any of the doses compared to control. IL-6 (p = 0.04) and IL-10 (p = 0.02) levels were significantly increased at the 5 µg dose 1 h post-exposure (Fig. 1E). KC/GRO and TNF-α were also significantly increased at 5 µg DHA (p = 0.007) 1 h post-exposure (Fig. 1E and G). Consistent with the results obtained from mice at 1 h post-DHA exposure, IFN-γ, IL-1β, IL-2, IL-5, IL-6, IL-12p70, and TNF-α levels were also found to remain unchanged compared to the saline group 24 h post-exposure (Fig. 1F). In contrast to the 1 h post-DHA exposure, IL-10 and KC/GRO levels had returned to the base levels at all doses at 24 h post-exposure (Fig. 1F and H). Overall, an increase in inflammatory cytokines was observed at 1 h post-exposure in DHA-exposed mice, and levels returned to baseline 24 h after exposure.

**Fig. 2:**
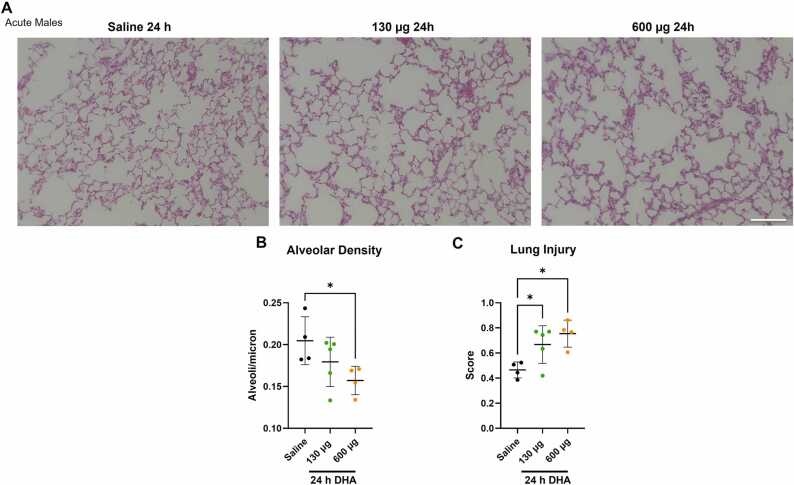
Acute exposure to DHA induced acute lung injury in male mice. Lung sections from male A/J mice were exposed to saline vehicle, 130, and 600 µg of DHA were examined 24 h after acute exposure. H&E staining was performed and imaged (A) to measure alveolar density (B) and acute lung injury (C). Scale bar is 200 µm. Mean ± SD of density or score are displayed. Statistical significance was determined by ANOVA with significance is displayed as follows: *p < 0.05, **p < 0.01, ****p < 0.0001.

The lungs of mice acutely exposed to 130 and 600 µg DHA were stained with H&E (Fig. 2A) to measure alveolar density and acute lung injury score 24 h post-exposure. These doses were selected as significant changes in the BALF cell count were found with these doses. A significant decrease in alveoli density was observed in mice exposed to 600 µg of DHA (Fig. 2B). This suggests damage to alveolar septa due to the release of proteases and elastases by the neutrophils infiltrating the lungs. In addition, the acute lung injury score was significantly increased in mice exposed to 130 and 600 µg DHA (Fig. 2C).

### 3.2 Sub-acute exposures to DHA-induced emphysematous and fibrotic lung changes

**Fig. 3:**
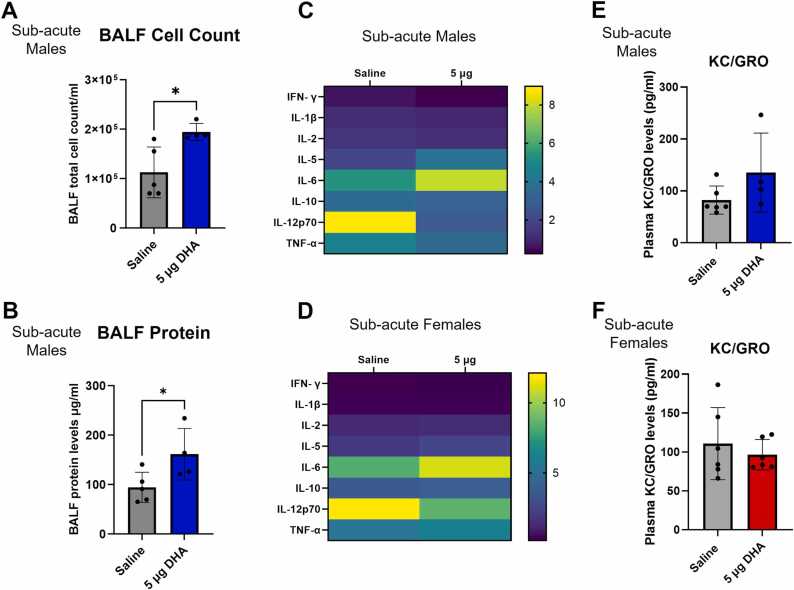
Sub-acute exposures to DHA promote inflammatory changes in male and female mice, and acute lung injury was observed in female mice. Male A/J mice were sub-acutely exposed to 5 µg of DHA for 2 weeks. BALF cell count (A) and protein levels (B) were measured in the vehicle control and DHA-exposed groups. Plasma from mice exposed sub-acutely to DHA was used to measure cytokine changes with the V-PLEX Proinflammatory panel. Cytokine levels in male A/J mice (C) and female A/J mice (D) are displayed by heat map with a separate KC/GRO graph for males (E) and females (F). Values are displayed as mean ± SD. Significance was determined by a Welch’s *t*-test and is displayed as follows: *p < 0.05.

Due to significant damage to lung function and architecture caused by exposure to 130 and 600 µg of DHA, sub-acute exposure to a lower dose of 5 µg DHA over 14 days was used to evaluate parameters of lung injury. To evaluate inflammatory changes in mice exposed to DHA, BALF cell count and protein levels were assessed in the sub-acute exposures after 2 weeks of 5 µg DHA exposure in male A/J mice (Fig. 3). BALF inflammatory cell counts and protein levels were significantly elevated in DHA-exposed mice (Fig. 3A and B). We also performed the inflammatory cytokine analysis to assess changes after the sub-acute exposures in males and females (Fig. 3C). In male A/J mice, IFN-γ and TNF-α were significantly decreased in the DHA-exposed group (p = 0.03; p = 0.04). IL-1β, IL-2, IL-5, IL-6, IL-10, IL-12p70, (Fig. 3C) and KC/GRO (Fig. 3E) remained unchanged compared to the saline group. In the female A/J mice, IFN-γ, IL-1β, IL-2, IL-5, IL-6, IL-10, IL-12p70, TNF-α, (Fig. 3D) and KC/GRO (Fig. 3F) were unchanged in DHA exposed group, consistent with the male A/J mice. There were sex-specific differences in the cytokine response to sub-acute dosing, with male mice showing more significant changes in these cytokines than the females.

**Fig. 4:**
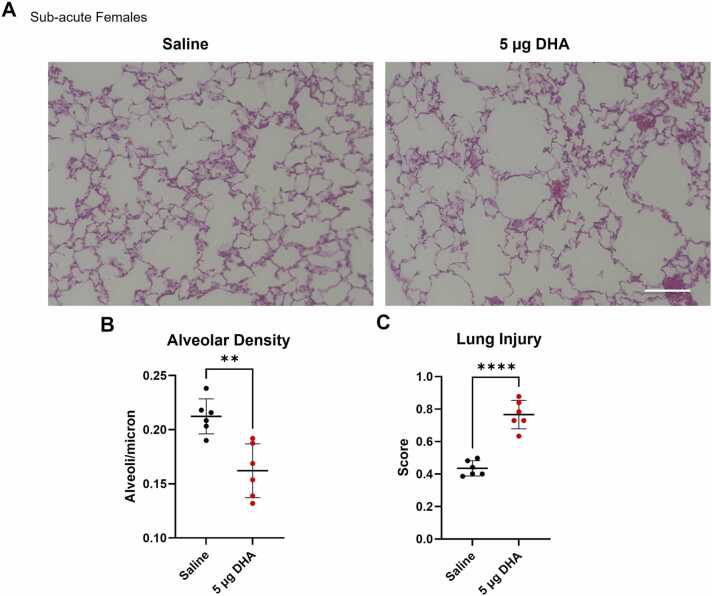
Sub-acute exposures to DHA promote acute lung injury in female mice. Female A/J mice were sub-acutely exposed to 5 µg of DHA for 2 weeks and H&E staining was performed on lungs (A). Alveolar density (B) and acute lung injury scores (C) were measured. Density and scores are displayed as mean ± SD. Significance was determined by a Welch’s *t*-test and is displayed as follows: **p < 0.01, ****p < 0.0001. Scale bar is 200 µm.

Next, we assessed female mice’s alveolar density and lung injury score after sub-acute exposures to 5 µg DHA by staining lungs with H&E stain (Fig. 4A). Decreased alveolar density was found in DHA-exposed mice compared to the vehicle saline group (Fig. 4B). This suggests damage to alveolar septa similar to the emphysematous obstructive lung phenotype seen in people with COPD due to the proteolytic damage to alveoli by proteases and elastases released by elevated levels of neutrophils in the lungs. We confirmed this effect by staining the lungs for the neutrophil myeloperoxidase (MPO) stain and observed a significant increase in sub-acutely DHA-exposed mice (Suppl Fig. 1). The analysis of lung histology also showed a significant increase in lung injury scores in mice exposed to DHA (Fig. 4C), further confirming lung damage by neutrophils.

**Fig. 5:**
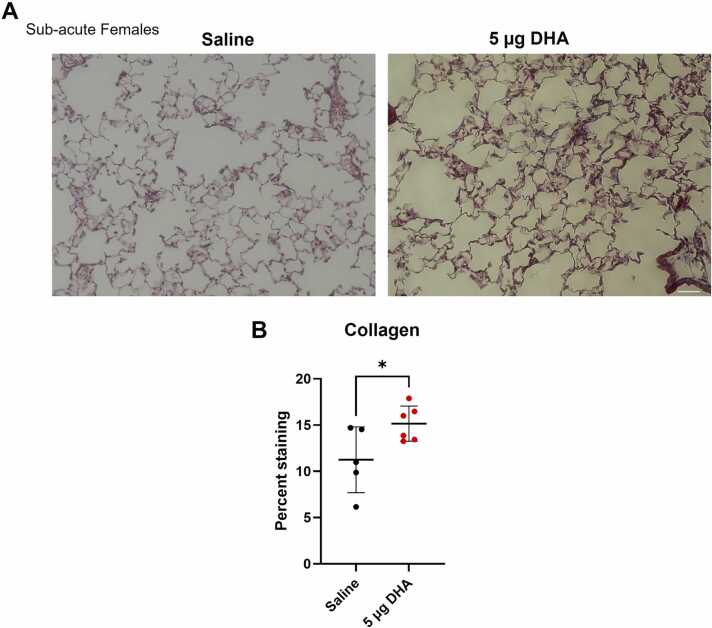
Sub-acute exposures to DHA increase collagen deposition. Masson’s Tri-chrome staining was performed on female A/J mouse lungs sub-acutely exposed to 5 µg of DHA for 2 weeks, and collagen staining was quantified. The graphs are displayed as mean ± SD. Significance was determined by a Welch’s *t*-test and is displayed as follows: *p < 0.05. Scale bar is 200 µm.

To further evaluate sub-acute lung changes, we stained the lungs of mice with Masson trichrome stain to measure the collagen deposition in the female mice (Fig. 5A). Data showed a significant increase in collagen deposition within the lungs after DHA exposure, indicating lung fibrosis (Fig. 5B). This indicates DHA exposure not only causes an obstructive emphysematous phenotype but also a fibrotic restrictive phenotype, indicative of a mixed lung disease.

**Fig. 6:**
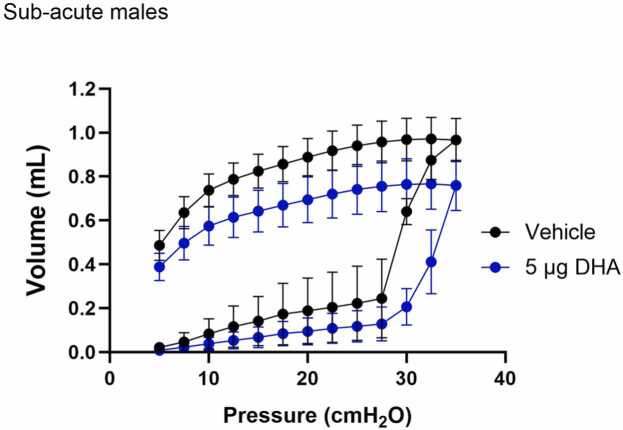
Lung function was altered after sub-acute exposures to DHA. The pressure-volume (PV) curve was measured using a FLEXIVENT machine. The graphs are displayed as mean ± SEM.

Finally, the lung function and mechanics were measured using FLEXIVENT (Fig. 6). The analysis of the PV curves indicated a slight decline in the lung volume as pressure increases in DHA-exposed animals, indicating a predisposition towards restrictive disorder. However, there were no robust changes in the pressure-volume curves post-DHA exposure due to the mixed (obstructive and restrictive) nature of the injury caused by DHA. The lung mechanics were further evaluated in mice by performing a methacholine challenge ranging from 0 to 40 mg/ml (Suppl Fig. 2). Total lung resistance (R_RS_) and compliance (E_RS_) of the respiratory system significantly increased in the DHA group at the highest methacholine dose (40 mg/ml) (Suppl Fig. 2A). Tissue damping (G), elastance (H), and Newtonian (central) airway resistance (R_N_) were also measured (Suppl Fig. 2B). No change was observed in R_N,_ while G and H significantly increased in the DHA group at the 10 and 40 mg/ml methacholine injection (Suppl Fig. 2B).

## 4 Discussion

The FDA has approved topical exposures to DHA. However, the advent of spray tanning in the late 1990s raised concerns about inhalation exposures to DHA through spray tanning applications. The FDA has issued warnings against inhalation and mucous membrane exposures during spray tanning, but the inhalation exposure effects of DHA are poorly understood [23]. DHA research has largely focused on skin or systemic models. Only one report has examined air-liquid-interface culture exposures with DHA. The air-liquid-interface cultures were acutely and continuously exposed to high concentrations of DHA every 7 days for up to 5 weeks [35]. Cilia beating frequency and MUC5AC (mucin 5AC) secretion initially decreased after exposure. However, no significant morphological changes were observed in DHA-treated cultures after 5 weekly exposures. They concluded sporadic exposures to DHA had transient toxic effects on human airway cultures [35].

Given the identification of DHA in the aerosol of e-cigarettes, the importance of understanding inhalation, particularly daily chronic exposures, has grown. We have previously characterized the cytotoxic and genotoxic effects of DHA across various systemic models, demonstrating DHA induces metabolic reprogramming, mitochondrial dysfunction, and replication stress [24], [25], [26], [27]. More recently, we have confirmed DHA is genotoxic to lung, liver, and cardiac cell models and demonstrated DHA induces chromosomal instability and mutagenesis [36].

With these *in vitro* findings, we sought to characterize acute and sub-acute exposures to DHA in A/J mice. One significant limitation to evaluating DHA exposure effects is the lack of quantitative data about the inhaled doses of DHA during spray tanning or e-cigarette use. We have addressed this limitation by using measured ranges of DHA from mass spectrometry studies, self-reported vaping behaviors, and puff topography reports [6], [8], [18], [19]. We conservatively selected our doses of DHA for testing with low, moderate, and high doses reflected by the 5, 130, and 600 µg doses selected. While these estimates may have significant errors, particularly from self-report bias, they are consistent with the European Union estimates for one-time exposures from spray tanning booths of 0.21–0.6 mg and represent a reasonable starting point [34].

We initially tested acute dosing in the male A/J mice at 1 and 24 h. The analysis of BALF from these animals showed a dose-dependent increase in lung leak within 1 h and infiltration of inflammatory cells into the lungs within 24 h post-DHA exposure (Fig. 1A and B). The quantification of cytokine levels revealed an increase in cytokines, including IL-6, IL-10, KC/GRO, and TNF-α at 1 h post-DHA exposure. The cytokine levels stabilized at 24 h, indicating an initial inflammatory response due to inhalation of DHA (Fig. 1C and D). We also observed lung injury and changes in alveolar density 24 h after one-time exposure to 130 and 600 µg DHA (Fig. 2). These results support DHA inducing adverse effects in the lungs acutely. Due to the uncertainty in inhaled doses, we evaluated the lowest 5 µg dose for sub-acute exposures.

Sub-acute dosing of male A/J mice revealed significant BALF protein and cell count changes (Fig. 3A and B). At sub-acute exposures, the male mice showed a significant reduction in IFN-γ and TNF-α levels in contrast to the elevation of inflammatory cytokines after acute exposures. Sustained exposures to DHA resulted in a mild inflammatory response with elevated levels of IL-6 observed in both sexes, though these levels were not significantly elevated (Fig. 3C and D). We also observed elevated IL-12p70 and TNF-α in the saline exposures compared to the DHA exposures in the male and female mice (Fig. 3D and F). Cytokine increases after saline exposure were shown previously in A/J and C57BL/6 J mice, though in that study, results were compared to the high induction of cytokines after lipopolysaccharide (LPS) exposures [37]. Given that we only observed low levels of cytokines induced by saline in our study, our results are consistent with this work and likely suggest differences in osmolarity between the compounds influence cytokine release [37], [38].

In general, we observed more significant changes in cytokine levels in the male mice at both acute and sub-acute doses. The female mice, only measured after sub-acute exposures, showed no significant changes in cytokine levels, suggesting sex-specific responses to DHA exposure (Fig. 3D and F). Further work is needed to understand the sex-specific differences. However, DHA is a carbohydrate and sex-specific differences in carbohydrate metabolism are noted across species [39], [40], [41], [42]. We have previously demonstrated that metabolic differences impact DHA responses [26], [27], [43]. Therefore, metabolic differences likely dictate DHA exposure effects.

We also confirmed that DHA induces lung injury in sub-acutely exposed female mice using immunohistochemistry. DHA-exposed female mice showed increased lung injury scores and reduced alveolar density due to alveoli rupture indicative of obstructive lung phenotype in lung emphysema (Fig. 4). Meanwhile, increased collagen deposition demonstrates a restrictive phenotype associated with fibrosis (Fig. 5). Similar effects were characterized in other environmentally induced chronic lung injuries, such as those with prolonged cigarette smoke or toxic gases [44], [45].

Functional changes in the lung were consistent with the histological findings. While the changes in PV loops are insignificant after the 2 weeks of sub-acute exposure due to the injury’s mixed nature (obstructive and restrictive), the methacholine challenge supports a trend of reduced respiratory function (Fig. 6 and Suppl. Fig. 2). Interestingly, our sub-acute exposure to DHA alone showed increased tissue dampening and response to methacholine consistent with e-liquid exposure composed of 70 % vegetable glycerin (VG) and 30 % propylene glycol (PG) with French vanilla to female C57BL/6 [17]. While a limitation in this study is the lack of lung function analysis in the female mice, we have demonstrated that DHA induces lung injury and a fibrotic phenotype in both male and female A/J at the low dose of 5 µg. These results suggest that DHA may contribute to adverse effects observed after exposure to aerosolized e-liquids.

These preliminary *in vivo* results extend the extensive *in vitro* findings that DHA induces harmful cellular effects. Future studies should increase the dose duration and incorporate the moderate dose to determine if there are dose-dependent effects. In cardiac models, low non-cytotoxic doses stimulate metabolic reprogramming to adapt to DHA as a fuel source; however, this adaption only occurs over a low dose range [27]. Understanding the dose response *in vivo* will be critical to understanding the contributions of DHA to adverse e-cigarette exposures.

## Funding

NIH/NIEHSR01 ES032450 supported NRG. HL is supported by R01 ES032450–04S1, AH is supported by CCTS TL1TR003106. SA is supported by U01 ES033265.

## CRediT authorship contribution statement

**Saurabh Aggarwal:** Writing – review & editing, Writing – original draft, Visualization, Methodology, Investigation, Funding acquisition, Formal analysis, Data curation, Conceptualization. **Jenna Hedlich-Dwyer:** Writing – review & editing, Writing – original draft, Methodology, Investigation, Formal analysis, Data curation. **Natalie R Gassman:** Writing – review & editing, Writing – original draft, Visualization, Methodology, Investigation, Funding acquisition, Formal analysis, Data curation, Conceptualization. **Hailey Levi:** Writing – review & editing, Methodology, Investigation, Data curation. **Lilly Underwood:** Writing – review & editing, Formal analysis, Data curation. **Juan Xavier Masjoan Juncos:** Writing – review & editing, Resources, Methodology, Investigation, Formal analysis, Data curation, Conceptualization. **Arlet Hernandez:** Writing – review & editing, Writing – original draft, Methodology, Investigation, Formal analysis, Data curation, Conceptualization.

## Declaration of Generative AI and AI-assisted technologies in the writing process

Generative AI was not used in the preparation or revision of this manuscript.

## Declaration of Competing Interest

The authors declare that they have no known competing financial interests or personal relationships that could have appeared to influence the work reported in this paper.

## Data Availability

Data will be made available on request.

## Appendix A Supplementary material

Supplementary material **Suppl** Fig. 1**. Myeloperoxidase staining lung tissues.** Female A/J mice were treated with either saline or 5 µg DHA sub-acutely for 2 weeks. Immunofluorescence was performed on lung tissue slides for myeloperoxidase (MPO), and the intensity was quantified. Fluorescence intensity was graphed as mean ± SEM of values. Scale bar is 500 µm. **Suppl** Fig. 2**. Physiological lung parameters were measured using FLEXIVENT.** Male A/J mice were exposed to either saline or 5 µg of DHA for 2 weeks. FLEXIVENT machine injected methacholine challenges to evaluate the resistance of the respiratory system (R_RS_), overall compliance of the respiratory system (E_RS_), tissue damping (G), tissue elastance (H), and resistance (R_N_). The values were graphed in GraphPad Prism as mean ± SEM. Significance displayed as follows: *p < 0.05, * *p < 0.01
