# Supplementary figures and images for: Inhalation exposure to dihydroxyacetone promotes lung injury and pulmonary fibrosis in A/J mice
Source: Toxicol Rep. 2024 Dec 18;14:101878. doi: 10.1016/j.toxrep.2024.101878 (PMC12223428; doi:10.1016/j.toxrep.2024.101878)

**A** Sub-acute Females

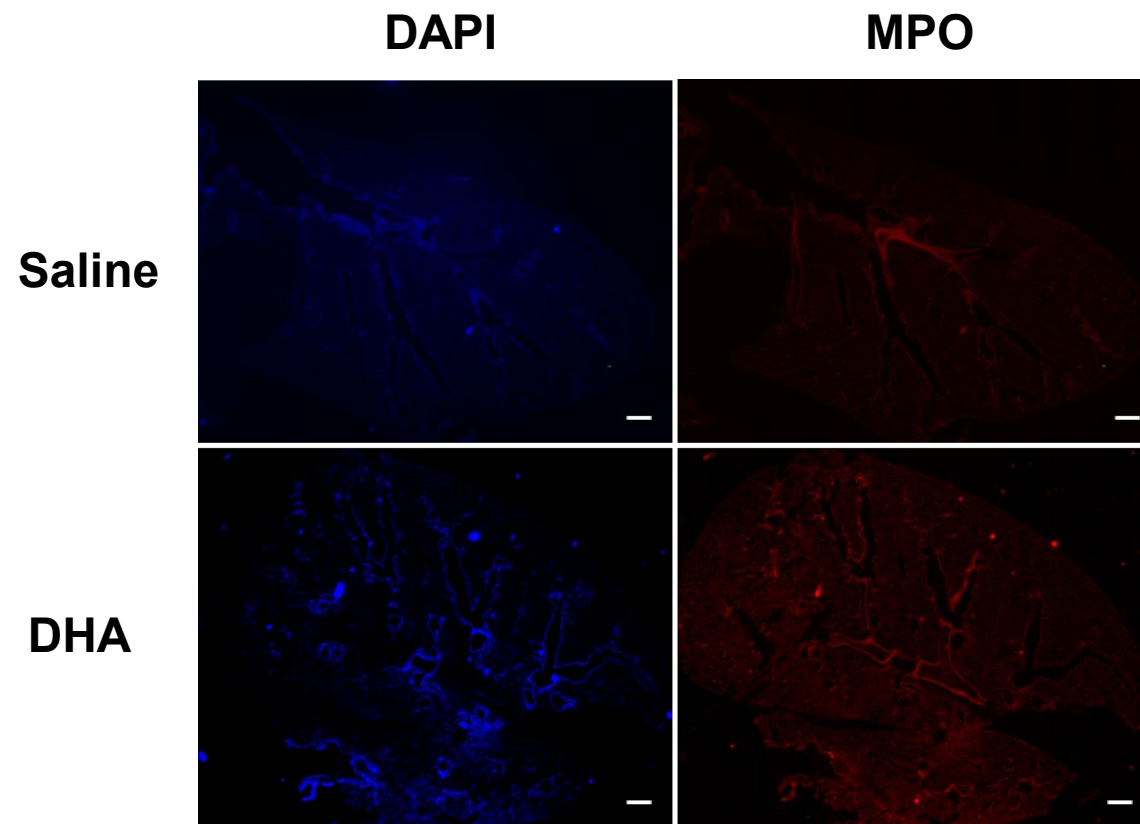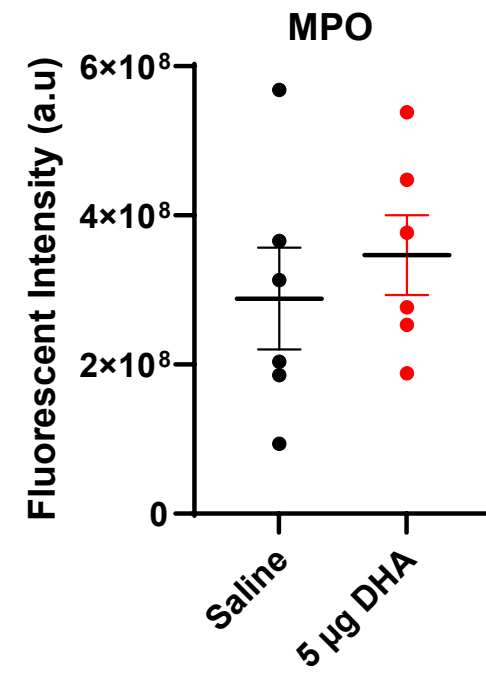

**A**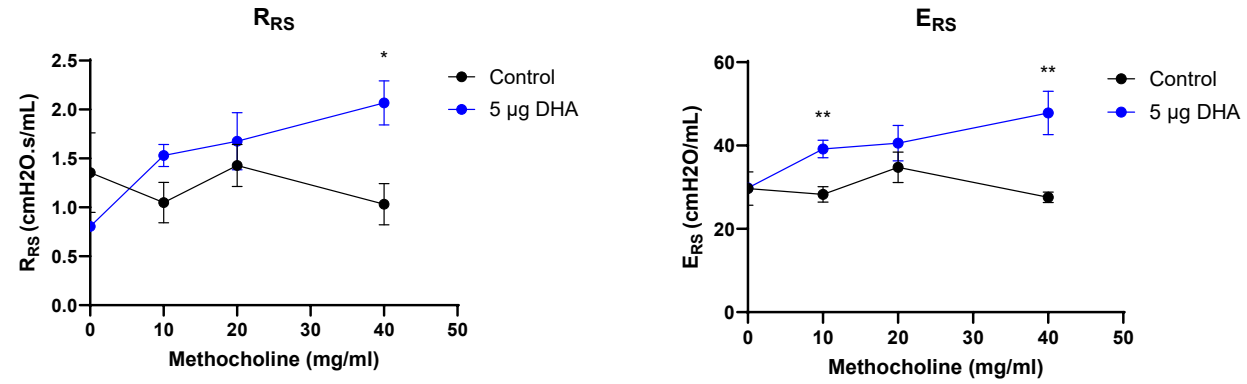**B**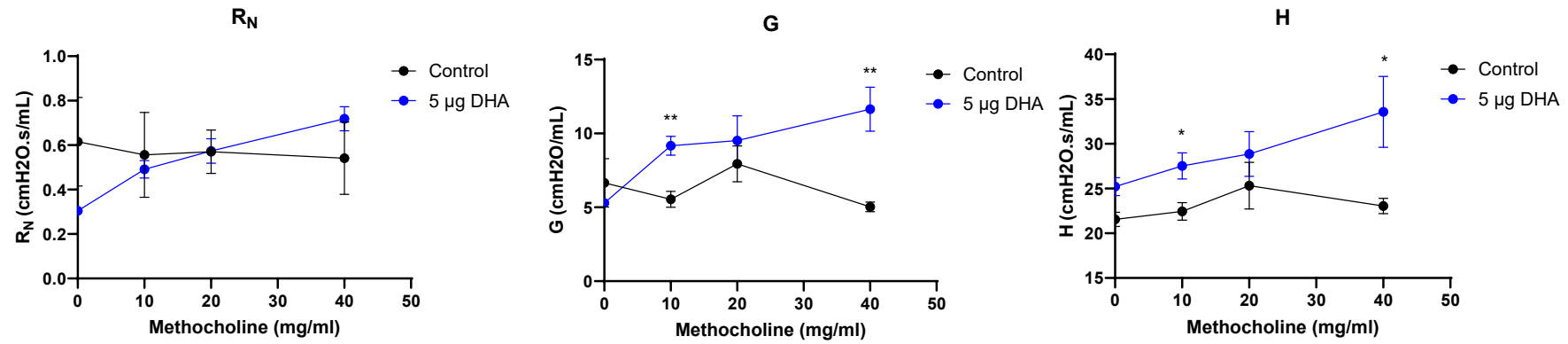

Supplement: Supplementary file 1 — Supplementary material Suppl Fig. 1. Myeloperoxidase staining lung tissues. Female A/J mice were treated with either saline or 5 µg DHA sub-acutely for 2 weeks. Immunofluorescence was performed on lung tissue slides for myeloperoxidase (MPO), and the intensity was quantified. Fluorescence intensity was graphed as mean ± SEM of values. Scale bar is 500 µm. Suppl Fig. 2. Physiological lung parameters were measured using FLEXIVENT. Male A/J mice were exposed to either saline or 5 µg of DHA for 2 weeks. FLEXIVENT machine injected methacholine challenges to evaluate the resistance of the respiratory system (RRS), overall compliance of the respiratory system (ERS), tissue damping (G), tissue elastance (H), and resistance (RN). The values were graphed in GraphPad Prism as mean ± SEM. Significance displayed as follows: *p < 0.05, * *p < 0.01 [file mmc1.pdf]
